# Endothelial-Leukocyte Interaction in Severe Malaria: Beyond the Brain

**DOI:** 10.1155/2015/168937

**Published:** 2015-09-30

**Authors:** Mariana C. Souza, Tatiana A. Padua, Maria G. Henriques

**Affiliations:** ^1^Laboratory of Applied Pharmacology, Farmanguinhos, Oswaldo Cruz Foundation, Avenida Brasil 4365, Manguinhos, 21040-900 Rio de Janeiro, RJ, Brazil; ^2^National Institute for Science and Technology on Innovation on Neglected Diseases (INCT/IDN), Center for Technological Development in Health (CDTS), Oswaldo Cruz Foundation (Fiocruz), Rio de Janeiro, RJ, Brazil

## Abstract

Malaria is the most important parasitic disease worldwide, accounting for 1 million deaths each year. Severe malaria is a systemic illness characterized by dysfunction of brain tissue and of one or more peripheral organs as lungs and kidney. The most severe and most studied form of malaria is associated with cerebral complications due to capillary congestion and the adhesion of infected erythrocytes, platelets, and leukocytes to brain vasculature. Thus, leukocyte rolling and adhesion in the brain vascular bed during severe malaria is singular and distinct from other models of inflammation. The leukocyte/endothelium interaction and neutrophil accumulation are also observed in the lungs. However, lung interactions differ from brain interactions, likely due to differences in the blood-brain barrier and blood-air barrier tight junction composition of the brain and lung endothelium. Here, we review the importance of endothelial dysfunction and the mechanism of leukocyte/endothelium interaction during severe malaria. Furthermore, we hypothesize a possible use of adjunctive therapies to antimalarial drugs that target the interaction between the leukocytes and the endothelium.

## 1. Introduction

Malaria is the most important parasitic disease worldwide. It is present in more than 100 countries, putting 1.2 billion people at risk and accounting for more than 800 thousand deaths each year [1, 2]. Cerebral malaria (CM) is the most severe form of malaria and is usually found in children under five years old [3]. Clinically, CM is defined by the identification of* P. falciparum* in peripheral blood, convulsions, and coma, after ruling out any other cause of coma such as meningitis [4, 5]. Pathological findings such as capillary congestion, production of proinflammatory cytokines, and adhesion of infected red blood cells (iRBC) to brain vasculature are responsible for cerebral complications associated with CM [6]. In some patients, a systemic illness called severe malaria (SM) is observed which is characterized by one or more peripheral organ dysfunctions as acute lung injury (ALI)/acute respiratory distress syndrome (ARDS) [7] and acute kidney injury [8, 9] and can be combined with cerebral malaria signals [10]. Some authors suggest that SM is due to pathological events such as parasitized erythrocytes, leukocyte adhesion to the organ microvasculature, systemic production of cytokines, and cytotoxic lymphocyte activation [11, 12]. Despite systemic activation, the leukocyte/endothelial cell interaction differs depending on the studied organ. Here, we discuss endothelial dysfunction during severe malaria and the mechanisms by which leukocytes adhere to the endothelium in distinct organs during this pathology.

## 2. Leukocyte-Endothelium Interaction during Cerebral Malaria

A main characteristic of brain physiology is the immune privilege conferred by the BBB to brain tissue [13]. However, the BBB composition, especially in the postcapillary venule, allows leukocyte diapedesis during nonmalarial brain injury [14, 15].

During human cerebral malaria, the importance of infected red blood cells adhesion to brain microvasculature is well established [5]. Necropsy of fatal cases of severe malaria shows the adhesion of iRBC in the venules and capillaries, causing congestion [6, 16, 17]. The mechanism of iRBC adhesion to brain microvasculature is well described and depends on expression of membrane proteins such as* P. falciparum* erythrocyte membrane proteins (PfEMP1) [18]. However, the leukocyte-endothelium interaction during human cerebral malaria is not completely clarified [12, 16, 19, 20]. Indeed, it is well established that both endothelium [21] and leukocyte [22, 23] are activated in patients diagnosed with CM; however, how they orchestrate the brain injury to develop CM is still not well understood.

Endothelium activation markers have been used in clinical studies to predict malaria severity [24, 25]. During CM, the endothelium can be activated by different mechanisms as the binding of soluble proteins present in host serum [24], direct contact with iRBC [6], and activation induced by parasite-derived molecules as hemozoin [26] and GPI [27]. Necropsy performed in fatal cases of CM showed increased expression of adhesion molecules on brain microvasculature [28] supporting the idea that the endothelium is able to promote leukocyte adhesion. Some studies show the presence of leukocyte in brain vasculature lumen [16] or in perivascular space [29], although there are no lines of evidence of the importance of leukocyte adhesion to brain vasculature in development of human CM. However, it cannot be ruled out considering the lack of knowledge in this issue [16, 20].

The interaction between leukocytes and endothelial cells during human CM could not depend on cell-cell contact. Instead, leukocytes and lymphocytes produce inflammatory mediators as TNF-*α* which activate endothelial cells [28, 30]. Endothelial activation induced by TNF-*α* accounts for many factors involved in development of CM [31] as increased iRBC adhesion [30], expression of leukocyte chemotactic factors [32] and, costimulated by iRBC, increases ICAM-1 expression that improve iRBC adhesion [30].

On the other hand, the adhesion of leukocytes to brain vasculature is often observed during experimental cerebral malaria [33, 34]. A recent report revealed that the majority of leucocytes accumulated in the brain during experimental severe malaria are monocytes. These cells are responsible for the recruitment of CD4^+^ and CD8^+^ T cells to the CNS vasculature [35]. However, in the absence of monocytes, T cells are still recruited to the brain to initiate experimental cerebral malaria [35]. Observation of the microvessels within the brains of live animals demonstrated the marginalization of leukocytes and platelets aggregates in postcapillary brain venules but not in capillaries of* P. berghei*-infected mice, showing that leukocytes do not accumulate in brains tissue but induce endothelium dysfunction, leading to vascular leakage, neurological signs, and coma [35, 36]. The role of adhesion molecules, especially ICAM-1, in the leukocyte/endothelium interaction to promote cerebral dysfunction during experimental severe malaria is controversial. The impairment of the ICAM-1/*β*2-integrin complex abolishes the development of cerebral dysfunction associated with* P. berghei* infection [36–38]. However, Ramos and colleagues deleted ICAM-1 in different cells and showed that only ICAM-1 expressed in leukocytes accounts for experimental severe malaria [39]. The authors speculated that because endothelial cells do not express ICAM-1 counter receptor, leukocytes, platelets, and iRBC aggregates occlude brain microvessels and promote cerebral malaria [39].

A new approach of leukocyte and endothelium interaction in brain during CM has been proposed through interaction between MHC class I molecules and CD8^+^ T lymphocytes. Recent studies regarding experimental CM show that the membranes of endothelial cells and iRBC fuse by trogocytosis, resulting in the expression of* Plasmodium* antigens [40]. Endothelial cells preferentially phagocytize merozoites and, via proteasome digestion, present plasmodial antigens by MHC class I molecules to CD8^+^ T lymphocytes, thus contributing to the adaptive immune response to* P. berghei* infection [41]. It is noteworthy that the same results were observed within* P. falciparum* phagocytosis by human endothelial cells [41]. However,* P. falciparum* phagocytosis by endothelial cells* in vivo* and its clinical relevance remain to be elucidated.

**Figure 1:**
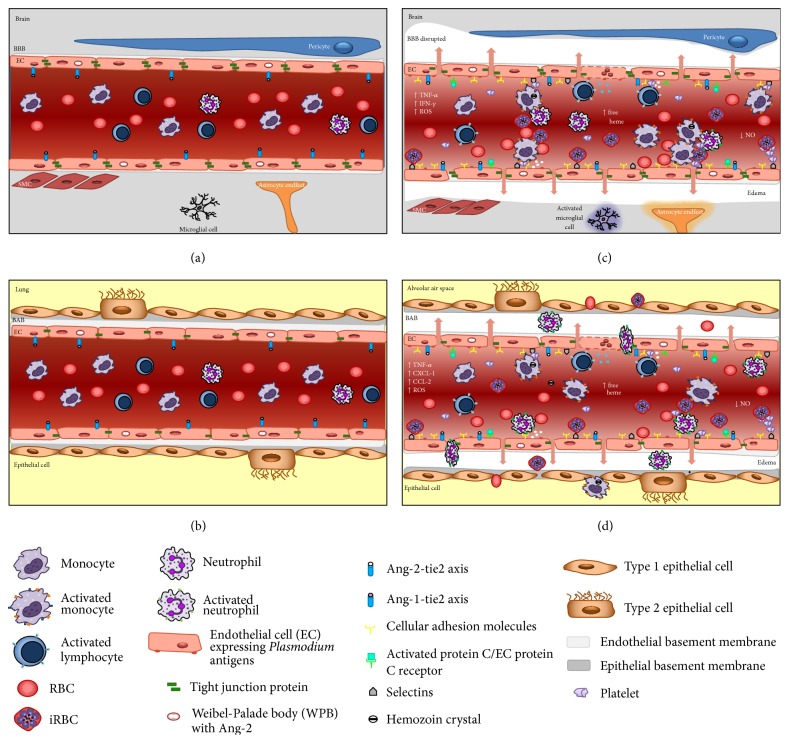
Blood barrier differences between brain and lung during malaria. (a) Cerebral microvasculature and (b) lung microvasculature without leucocytes attached in postcapillary venules and EC expressing Ang-1, under physiological conditions. (c) During severe malaria, we observe production of proinflammatory cytokines, increase of cellular adhesion molecules expression, release of Ang-2, decrease of NO, and adhesion of iRBC and leukocytes (mainly mononuclear cells) to brain vasculature leading to capillary congestion, BBB dysfunction, and edema. Such events activate the subjacent tissue (microglial cells and astrocytes). (d) Acute lung injury (ALI) and acute respiratory distress syndrome (ARDS) associated with malaria. The augment of inflammatory cytokines and chemokines, release of Ang-2, and decrease of NO are responsible for activation of EC that increases the expression of cellular adhesion molecules allowing the margination and infiltration of iRBC, leucocytes, and platelets into blood vessels, interstitial tissue, and consequently alveolar air space. BBB: blood-brain barrier; BAB: blood-air barrier; EC: endothelial cell; ROS: reactive oxygen species; SMC: smooth muscle cell.

Overall, microvascular congestion observed in both human and experimental CM leads to severe cerebral endothelial damage, resulting in the breakdown of the BBB mainly at the level of postcapillary venules [16, 29, 31, 42]. The postcapillary venule BBB (Figure 1) is functionally distinct from other BBB areas and is in direct contact with the perivascular space [42]. In light of the new findings concerning brain anatomy in which the authors described the presence of lymphatic vessels in direct contact with the perivascular space in the central nervous system, in the next few years, the dynamics of the interaction between leukocytes and the endothelium during cerebral malaria will likely be unveiled [43].

## 3. Leukocyte-Endothelium Interaction in the Lung during Malaria

The brain is not the only organ affected during severe malaria. Twenty percent of patients diagnosed with severe malaria develop acute lung injury (ALI) and acute respiratory distress syndrome (ARDS) [7, 9]. ALI/ARDS is a syndrome derived from pathological conditions such as sepsis and traumatic brain injury. ALI/ARDS diagnosis includes the identification of respiratory failure, diffuse alveolar damage, and inflammatory infiltration in lung tissue [44]. Necropsy in fatal cases of severe malaria revealed that patients present classical symptoms of ALI, including pulmonary edema, pulmonary capillary congestion, thickened alveolar septa, marked inflammatory response in lung tissue, and macrophages in the lumen of the pulmonary capillaries [11]. Murine experimental models of severe malaria also present pulmonary pathology such as edema, cell infiltration, tissue damage, and lung mechanical impairment [45–48]. Furthermore, the lung appears to be a large reservoir of metabolically active parasites, as described in an elegant study by Lovegrove et al. who evaluated the transcriptional responses to* Plasmodium* in different organs [49].

The lung vasculature in malaria infection is essential to initiate the* Plasmodium* cycle within the host. When merozoites leave the liver, they are located inside host-derived buds named merosomes, whose membranes are disrupted within the pulmonary capillary beds to allow merozoites to reach the erythrocytes [50, 51]. The close contact between infected erythrocytes and pulmonary endothelial cells triggers a remarkable inflammatory response 24 h after infection, characterized by intense inflammatory cell infiltration as well as the production of proinflammatory cytokines and chemokines in lung tissue that persists for at least five days after infection [45–47]. The quantity of parasites in lung tissue defines the extent of chemokine production in lung tissue [52]. Chemokines such as CCL2, CXCL1, and CCL5 are produced in lungs during experimental malaria and are correlated with macrophage and neutrophil accumulation in pulmonary tissue [45, 53, 54]. Intravital studies in lungs of* Plasmodium*-infected mice reveled edema formation and the migration of monocytes and neutrophils to lung tissue [37]. However, due to the technical limitations in studying leukocyte mobility within the lung [55], until now, there have been no available data on the dynamics of leukocytes and lung endothelium during malaria-triggered ALI [37]. Indeed, lung endothelial cells are activated during malaria infection and express adhesion molecules. P-selectin, in addition to L- and E-selectin, is part of a family of calcium-dependent (C-type) lectins whose activation induces the expression of *β*2-integrins and consequent leukocyte arrest in the vasculature [56, 57]. P-selectin is expressed in both lung and brain endothelium during experimental malaria. This molecule mediates leukocyte rolling in brain microvessels of* P. berghei*-infected mice; however, it is not essential for development of experimental cerebral malaria signals [58]. On the other hand, the monocyte/macrophage accumulation in lungs of* P. berghei*-infected mice depends on the expression of ICAM-1 [52, 59], while ICAM-1 expression in the brains of infected mice does not account for leukocyte adhesion [60]. It is interesting to note that while inflammatory cell infiltration in cerebral tissue was not observed in the brain, neutrophil and macrophage infiltration is frequently observed in pulmonary interstitial lung tissue during malaria [45]. Indeed, differences in the blood-brain barrier and blood-air barrier tight junction constitution of the brain and lung are responsible for this phenomenon (Figure 1).

The morphological and biochemical differences between lung and brain endothelial cells account for the distinct inflammatory responses in both organs. Despite both endothelial cell types containing nonfenestrated endothelium, brain endothelial cells present fewer caveolae and are richer in tight junctions than lung endothelium [61, 62]. The lung endothelial bed is rich in adherens junctions and P-selectins and allows leukocyte transmigration by paracellular and transcellular pathways [61, 62]. Endothelial cells from lung tissue can be activated by VEGF [4], TNF-*α* [63], LPS [64], and* P. falciparum* infected erythrocytes, resulting in the reorganization of their junctional proteins [63]. In addition to inflammatory mediators and pathogen-associated molecular pattern (PAMP), the leukocyte contact also contributes to endothelial cell reorganization, triggering a dephosphorylation cascade followed by the endocytosis of VE-cadherins, which support leukocyte transmigration through lung endothelial cells [65]. Of note, in most organs, leukocyte transmigration happens almost exclusively in postcapillary venules. However, in the lung leukocyte transmigration occurs in capillaries of the blood-air barrier which are surrounded by epithelium forming alveoli [61].

In addition to the direct interaction between leukocytes and the endothelium described earlier, leukocytes can bind platelets and then adhere to the endothelium. Piguet and colleagues showed that platelet and mononuclear cell trapping occurs in the lungs of* P. berghei*-infected mice [66]. In addition, the authors observed that the impairment of platelet activation decreased leukocyte adhesion to the lung vasculature of* P. berghei*-infected mice [66]. The stimulation of the receptor P2Y_1_ but not P2Y_12_ on platelets induces the downstream activation of the RhoA pathway, resulting in platelet/leukocyte aggregation and migration to the lung [67]. In addition, platelets also contribute to the leukocyte/endothelium interaction by releasing microparticles. Neutrophils stimulated with platelet-derived microparticles increased the expression of *α*M integrin and adhered to pulmonary endothelial cells via ICAM-1 [68].

The study of leukocyte/endothelium interactions within the lung during malaria is limited but extremely important. Mice depleted of neutrophils showed reduced malaria associated ALI and delayed mortality [38], suggesting that further studies are necessary to show the mechanism of the leukocyte/endothelial interaction in the lung during severe malaria.

## 4. Leukocyte/Endothelium Interaction during Malaria as a Target for Treatment

In accordance with the findings presented above both in human and animals, the leukocyte/endothelium interaction plays a role in the development of pathogenesis of severe malaria particularly in malaria-induced ALI [9, 12, 45, 47]. In fact, lung dysfunction triggered in both human and experimental malaria shares similarities with lung mechanics impairment, pulmonary edema, production of inflammatory cytokines, and inflammatory cells infiltration in lung tissue [9, 45]. Furthermore, the inflammatory response persists even after the host is cured of infection [10, 69, 70] (unpublished data), which suggests that modulation of inflammatory response in addition to antimalarial therapy would be helpful to patient outcome [71]. The leukocyte-endothelium interaction is not the most important factor regarding development of human cerebral malaria pathogenesis; however, it should not be neglected as actor in severe malaria-induced organ dysfunction.

**Figure 2:**
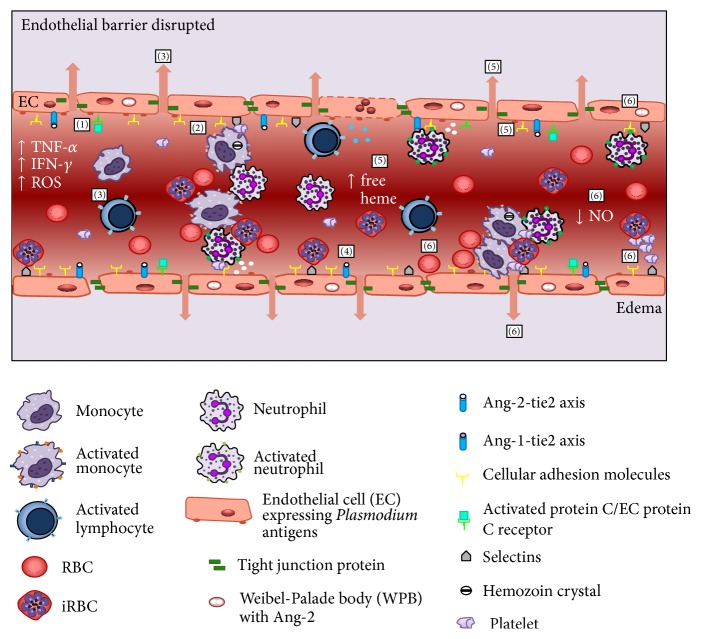
Targets of adjuvant therapies during malaria. Scheme showing several approaches that have been investigated aiming at modulation of malaria-induced inflammatory response. EC: endothelial cell; ROS: reactive oxygen species; SMC: smooth muscle cell. (1)* Activated protein C* binds to protein C receptor in activated EC cells decreasing the expression of adhesion molecules. (2)* Statins* decrease the production of chemokine and diminished the adhesion of leukocytes to brain microvasculature. (3)* Sphingosine-1-phosphate (S1P)* decreases the numbers of lymphocytes in brain vasculature and stabilizes the tight junction protein ZO-1 in brains. (4)* Neuregulin-1* and* bone marrow mesenchymal stromal cells* induce Ang-1, which promotes stabilization of EC tight junctions, EC desensitization to TNF-*α*, and downregulation of ICAM-1 and VCAM-1. (5)* Lipoxin A*_*4*_ decreases production of proinflammatory cytokines, impairs EC activation, and inhibits the expression of cellular adhesion molecules involved in leukocyte adhesion by stimulating the activity of HO-1, which catabolizes free heme. (6)* L-Arginine* or* inhaled NO (iNO)* reduces pulmonary edema and, in addition, decreases cytoadherence of iRBC, hemorrhagic foci, and leukocyte and platelets adherence to brain vasculature by inhibiting of WPB exocytosis that impairs the release of Ang-2 and inhibiting TNF-*α* production and procoagulant activity of endothelial cells.

Recently, Frosch and John suggested that an adjunctive therapy that impaired the inflammatory response induced during malaria should be combined with antimalarial drugs [72]. Several approaches have already aimed at the modulation of the malaria-induced inflammatory response.  Figure 2 illustrates several potential targets described in the literature. Patients diagnosed with severe malaria have been treated with modulators of TNF-*α* production [73], CD36 expression [74, 75], NO precursors [70, 76], or adhesion of iRBC to vasculature [77] and presented decreased inflammation scores when compared to a placebo treated group. Despite evidence suggesting that the modulation of leukocyte and endothelial activation supports the outcome of severe malaria, it is not clear whether an adjunctive therapy targeting the leukocyte/endothelium interaction would predict patient outcome. It is worth noting that the most important class of antimalarial drug to treat severe malaria is artemisinin and its derivatives [78], which also have immunomodulatory activities in pathologies such as microbial infections, tumor growth, and inflammatory diseases [79–82]. Our group demonstrated that, in addition to its antimalarial properties, artesunate exerted a protective effect against severe malaria via its immunomodulatory properties by inhibiting endothelial cell activation, NF-*κ*B nuclear translocation, and the subsequent expression of ICAM-1 [83].

Srinivas and colleagues studied the effect of treatment with activated protein C on a patient with severe malaria coinfected with leptospirosis and observed a rapid outcome [84]. The binding of activated protein C to endothelial cell protein C receptor in activated endothelial cells avoided NF-*κ*B p65 phosphorylation and induced AKT signaling, which decreased the expression of adhesion molecules on the endothelial cell surface [18, 85]. Thus, activated protein C in malaria would increase endothelial barrier integrity, induce antiapoptotic pathways, and decrease adhesion molecule expression [86]. Other modulators of endothelial functions have been used to evaluate malaria outcome in humans and experimental models. Studies in which* P. berghei*-infected mice were treated with statins, a class of drugs that inhibit the rate-limiting step in cholesterol synthesis and that show pleiotropic effects, demonstrated that statins decreased the production of chemokines [87] and decreased the adhesion of leukocytes to the brain microvasculature [88] probably by inhibiting the binding site of LFA-1 on leukocytes [89]. Accordingly,* in vitro* treatment of human endothelial cells with statins followed by stimulation with* P. falciparum*-infected erythrocytes decreased the expression of adhesion molecules, suggesting that statins could exert an antiadhesive role in the treatment of severe malaria [90]. Statins have not been tested in clinical trials for malaria adjunctive treatment. However, statins diminished the risk of sepsis-related mortality in patients, probably by decreasing the inflammatory response triggered during sepsis [91].

The endothelial barrier stabilizer sphingosine-1-phosphate (S1P) also rescued mice from severe malaria by decreasing the numbers of CD8^+^, CD4^+^, and CD45^+^ cells in the brain vasculature of* P. berghei*-infected mice, likely decreasing ICAM-1 expression and stabilizing the tight junction protein ZO-1 in brains [36, 92]. Transfection of bone marrow mesenchymal stromal cells and administration of S1P and other endothelial barrier stabilizers such as neuregulin-1 induce the endogenous Ang-1 anti-inflammatory pathway, which promotes decreased vascular permeability by stabilizing endothelial cell tight junctions, endothelial cell desensitization to TNF-*α*, and downregulating ICAM-1 and VCAM-1. These Ang-1 actions result in decreased leukocyte/endothelial interaction and, consequently, host outcome [4, 93–97].

Another family of lipids has been studied for its anti-inflammatory activity during severe malaria. Lipoxins (LX) are products of arachidonic acid metabolism and are produced through sequential lipoxygenase activity following cell-cell interactions in the inflammatory milieu (reviewed by [98]). The interaction of LXA_4_ and its receptor ALX has anti-inflammatory and proresolving activity in inflammatory models such as allergic airway inflammation [99] and autoimmune diseases [100] by reducing leukocyte adhesion to endothelial cells [101]. The administration of LXA_4_ improved survival in* P. berghei*-infected mice by decreasing the production of proinflammatory cytokines but not the accumulation of CD8^+^/IFN-*γ* ^+^ cells in brain tissue [102]. In addition to LXA_4_ impairment of leukocyte activation, the mechanism of action of LXA_4_ on endothelium during severe malaria was recently disclosed by intravital studies of the microvasculature of* P. berghei*-infected mice. The authors showed that treatment with LXA_4_ did not modulate leukocyte adhesion to the brain vasculature or decrease the expression of *β*2-integrin in leukocytes (unpublished data). On the other hand, treatment with LXA_4_ impaired endothelial activation during severe malaria and restored the blood flow in brains of* P. berghei*-infected mice [33]. The authors also showed that LXA_4_ exerted its effects by stimulating the activity of heme oxygenase 1 (HO-1), an isoenzyme that catabolizes free heme released under pathological conditions, especially in pathologies such as malaria which are associated with intravascular hemolysis [33]. HO-1 upregulation helps maintain BBB integrity under pathological conditions [103]. During the inflammatory response, HO-1 inhibits the expression of several adhesion molecules involved in leukocyte adhesion to endothelial cells [104, 105]. During experimental severe malaria, HO-1 is differentially regulated in certain tissues at different stages of* Plasmodium* life cycle [106, 107]. Furthermore, HO-1 production in brain tissue is associated with mouse survival, decreased cerebral edema, and decreased leukocyte adhesion to brain vasculature [106].

In hemolytic disorders such as malaria, low bioavailability of NO is observed, as free hemoglobin is a potent scavenger of this gaseous molecule [108]. Therefore, the administration of L-arginine or inhaled NO (iNO) has also been tested as adjunctive therapy in the treatment of severe malaria [4, 76]. Yeo and collaborators showed that impaired endothelial NO production occurred in severe malaria in both children and adults, supporting the idea that further trials of drugs that led to increased endothelial NO bioavailability could attenuate severe malaria symptoms [109]. Studies in which severe malaria patients were treated with inhaled nitric oxide demonstrated that NO reduced pulmonary edema in patients with malaria-derived ALI and decreased pulmonary capillary pressure through selective vasodilatory effects on postcapillary venules [110]. Thus, in severe malaria, nitric oxide is hypothesized to promote vascular quiescence, decrease cytoadherence of parasitized erythrocytes to the microvascular endothelium as a critical mediator of VEGF and Ang-1, and dampen inflammatory responses and thrombosis [4]. Nitric oxide (NO) is a short-lived free radical formed from L-arginine conversion that is involved in many important biological functions including neurotransmission, immune system, cytokine modulation platelet inhibition, vascular homeostasis, and regulation of hematopoiesis [111]. Its production occurs through three different NO synthase (NOS) enzyme isoforms: neuronal NOS (nNOS or NOS1), inducible NOS (iNOS or NOS2), and endothelial NOS (eNOS or NOS3) [111]. The constitutive isoforms (neuronal and endothelial) are calcium/calmodulin dependent and permanently active, generating low concentrations of NO. The inducible isoform (iNOS) is only expressed when its transcription is activated by a variety of cytokines, growth factors, and inflammatory stimuli on target cells, leading to the release of high levels of NO [112]. In experimental severe malaria, treatment with exogenous NO (NO donor dipropylenetriamine NONOate, DPTA-NO) showed improved pial blood flow, diminished hemorrhagic foci, and reduced leukocyte and platelet adherence to the brain vasculature [113, 114]. The authors hypothesize that NO attenuates malaria symptoms by (a) inhibition of Weibel-Palade body exocytosis and the consequent release of Ang-2 and increase in Ang-1 expression; (b) decreasing the endothelial expression of ICAM-1 and VCAM-1; (c) inhibiting TNF-*α* production; (d) inhibiting the procoagulant activity of endothelial cells; and (e) decreasing intravascular platelet aggregation [108, 110, 111].

Indeed, pathophysiological phenomena experienced during experimental severe malaria are not fully translated to human severe malaria [115–118]. Therefore, further studies should be performed before initiating clinical studies of immunomodulatory drugs as adjunctive therapy for severe malaria.
